# Exposure–response analysis using time-to-event data for bevacizumab biosimilar SB8 and the reference bevacizumab

**DOI:** 10.3389/fphar.2023.1288308

**Published:** 2024-01-16

**Authors:** Suemin Park, Jin Ah Jung, Sungil Ju, Hyeong-Seok Lim

**Affiliations:** ^1^ Asan Medical Center, Department of Clinical Pharmacology and Therapeutics, University of Ulsan College of Medicine, Seoul, Republic of Korea; ^2^ Asan Medical Center, Department of Medical Science, Asan Medical Institute of Convergence Science and Technology, University of Ulsan College of Medicine, Seoul, Republic of Korea; ^3^ Samsung Bioepis Co., Ltd., Incheon, Republic of Korea

**Keywords:** bevacizumab, biosimilar, non-small-cell lung cancer, time-to-event modeling, exposure–response analysis, simulation

## Abstract

**Purpose:** This analysis aimed to characterize the exposure–response relationship of bevacizumab in non-small-cell lung cancer (NSCLC) and evaluate the efficacy of SB8, a bevacizumab biosimilar, and Avastin^®^, the reference bevacizumab sourced from the European Union (EU), based on the exposure reported in a comparative phase III efficacy and safety study (EudraCT, 2015-004026-34; NCT 02754882).

**Materials and methods:** The overall survival (OS) and progression-free survival (PFS) data from 224 patients with steady-state trough concentrations (C_ss,trough_) were analyzed. A parametric time-to-event (TTE) model was developed using NONMEM^®^, and the effects of treatments (SB8 and bevacizumab-EU) and patient demographic and clinical covariates on OS and PFS were evaluated. Simulations of median OS and PFS by bevacizumab C_ss,trough_ were conducted, and concentrations required to achieve 50% and 90% of the maximum median TTE were computed.

**Results:** A log-logistics model with C_ss,trough_ best described the OS and PFS data. Treatment was not a predictor of the hazard for OS or PFS. Simulations revealed steep exposure–response curves with a phase of rapid rise before saturating to a plateau. The median C_ss,trough_ values of SB8 and bevacizumab-EU reported from the clinical study were on the plateaus of the exposure–response curves. The concentrations required to achieve 50% and 90% of the maximum effect were 82.4 and 92.2 μg/mL, respectively, for OS and 79.7 and 89.1 μg/mL, respectively, for PFS.

**Conclusion:** Simulations based on the constructed TTE models for OS and PFS have well described the exposure–response relationship of bevacizumab in advanced NSCLC. The analysis demonstrated comparable efficacy between SB8 and bevacizumab-EU in terms of OS and PFS based on their exposure levels.

## Introduction

SB8 is a biosimilar of the reference biologic bevacizumab (Avastin^®^; Roche), a recombinant humanized immunoglobulin G1 monoclonal anti-vascular endothelial growth factor (VEGF) antibody. Bevacizumab inhibits VEGF-induced tumor angiogenesis and suppresses tumor growth by binding to and neutralizing the biological activity of the VEGF (Ferrara et al., 2004; Roche Registration GmbH, 2023). Bevacizumab is used in combination with chemotherapy to treat various solid tumor types. Its therapeutic indications vary slightly across countries. In the European Union (EU), bevacizumab is authorized for the treatment of metastatic colorectal cancer, metastatic breast cancer, non-small-cell lung cancer (NSCLC), metastatic renal cell carcinoma, epithelial ovarian cancer, fallopian tube cancer, primary peritoneal cancer, and cervical cancer (Roche Registration GmbH, 2023).

SB8 was approved in the EU in 2020/2021 (Aybintio^®^/Onbevzi™) for the same cancer types as for Avastin^®^, based on the demonstration of comparable structural, functional, non-clinical, clinical pharmacokinetic (PK), efficacy, and safety profiles to the reference bevacizumab. The product subsequently received approval in the Republic of Korea (Onbevzi™), in Canada (Aybintio^®^) in 2021, and in Taiwan (Onbevzi™) in 2022 (Taiwan Food and Drug Administration, 2018; IPPR, 2021; Samsung Bioepis NL B.V, 2023b; Samsung Bioepis NL B.V, 2023a; Samsung Bioepis Co., Ltd., 2023). Analytical testing showed similarity between SB8 and the reference bevacizumab in terms of physicochemical properties (e.g., structure, biological activity, purity, and stability), and non-clinical testing showed functional similarity and *in vivo* pharmacological/toxicological activity between the two drug products. In support of establishing clinical similarity, two clinical studies were undertaken to compare the PK, efficacy, and safety of SB8 with its reference bevacizumab. In a phase I, randomized, single-dose, three-arm comparative PK study of healthy volunteers, the PK similarity of SB8 to both EU-sourced bevacizumab (bevacizumab-EU) and United States (US)-sourced bevacizumab (bevacizumab-US) was demonstrated, with the 90% confidence interval (CI) of the test-to-reference geometric mean ratio for the area under the serum drug concentration–time curves, as well as the maximum concentration, falling between 80% and 125% (Shin et al., 2020). In addition to the comparative phase I study, a phase III, randomized, double-blind, multicenter comparative study was conducted to evaluate the efficacy, safety, PK, and immunogenicity of SB8 compared with bevacizumab-EU in patients with metastatic or recurrent NSCLC. The study showed equivalence in the best overall response rate risk ratio, with comparable safety, PK, and immunogenicity profiles between SB8 and bevacizumab-EU (Reck et al., 2020). Upon the product’s approval, network meta-analysis, which combines direct and indirect evidence for the simultaneous comparison of multiple medicinal products, has been conducted, showing the similarity of SB8 to the reference product and other bevacizumab biosimilars (Xu et al., 2022).

The nature and scope of these studies performed during the development of SB8 offer a glimpse into the regulatory framework of biosimilar products, which is quite different from that associated with the development pathway for novel biologic drugs. The focus of the biosimilar development program is not to establish patient benefit *per se* but to demonstrate comparability at the analytical, functional, and clinical levels, as the development of biosimilars relies on existing scientific knowledge about the safety and effectiveness of the approved reference products (Weise et al., 2012). Nonetheless, conceptually, PK/PD (pharmacodynamic) modeling and simulation during biosimilar development can play a role similar to its role in the development of novel therapeutic proteins (Dodds et al., 2013; Wang et al., 2019). Pharmacometric analysis can provide insights into the exposure range that would be sensitive enough to assess clinically meaningful differences between biosimilars and reference products and can optimize study designs accordingly. However, such a similarity approach should be preceded by an understanding of the well-established exposure–response relationship of reference biologics and any variability associated with treatment.

As with most targeted protein therapeutics in oncology, patient responses may vary following treatment with bevacizumab in combination with chemotherapy. Multiple factors can contribute to this inter-patient variability, including, but not limited to, PK/biodistribution, drug interactions, tumor burden, and tumor heterogeneity (Symeonides and Gourley, 2015; Chakrabarti and Michor, 2017; Qi and Cao, 2023). The total plasma or serum VEGF concentration is used as an *in vivo* marker, and to date, its true association with the efficacy of bevacizumab for treating cancer in humans is uncertain, as the results of studies assessing the predictive value of serum VEGF levels for treatment outcomes have been not consistent (Presta et al., 1997; Bernaards et al., 2010; Tang et al., 2016). Aiming to find biomarkers, several studies have investigated the exposure–response relationship of bevacizumab, exploring trough concentrations associated with a threshold for treatment efficacy (Nugue et al., 2013; Caulet et al., 2016; Akbulut et al., 2018; Papachristos et al., 2020), and most of the research has focused on metastatic colorectal cancer, with little to no published data from investigations of bevacizumab’s exposure–response relationship in the treatment of advanced NSCLC.

Therefore, our modeling and simulation focused on the exposure–response modeling of bevacizumab, specifically for its use in the treatment of metastatic or recurrent NSCLC. This study aimed to 1) characterize the exposure–response relationship of SB8 and bevacizumab-EU in patients with advanced NSCLC and 2) evaluate the therapeutic efficacy of SB8 and bevacizumab-EU based on the exposure of each drug product.

## Materials and methods

### Study population

The clinical data used in this analysis were collected from 224 patients in a phase III comparative efficacy and safety study (SB8-G31-NSCLC) comparing SB8 (bevacizumab biosimilar) and the reference bevacizumab in the treatment of metastatic or recurrent non-squamous NSCLC (Reck et al., 2020). The patients in this study were randomized to receive SB8 or the reference bevacizumab (Avastin^®^, bevacizumab-EU), each at 15 mg/kg intravenously (IV), with paclitaxel 200 mg/m^2^ and carboplatin area under the curve 6, every 3 weeks for 4–6 cycles of the induction period. The tumor size was assessed by radiographic imaging, and patients with complete response, partial response, or stable disease as per the Response Evaluation Criteria in Solid Tumors (RECIST) after the induction period were given maintenance therapy with SB8 or bevacizumab-EU, each at 15 mg/kg IV every 3 weeks until progressive disease, unacceptable toxicity, death, or the end of the study, whichever occurred first. Trough serum concentrations of SB8 and bevacizumab-EU were assessed by pre-dose sampling before IV infusion at cycles 1, 3, 5, and 7. The exposure–response analysis included the overall survival (OS) and progression-free survival (PFS) assessed by blinded independent central review. Data from patients with available steady-state trough concentrations for SB8 (*n* = 100) and bevacizumab-EU (*n* = 124) measured just before cycle 5 or cycle 7 were included in the analysis.

Study SB8-G31-NSCLC was registered at EudraCT (2015-004026-34) and ClinicalTrials.gov (NCT 02754882). The study was in compliance with the Declaration of Helsinki, International Conference on Harmonization (ICH), and Good Clinical Practice (GCP) guidelines, as well as applicable local regulations. The protocol was approved by the local ethics committees at each study center, and informed consent was obtained from all patients before enrolling in the trial.

### Parametric TTE model development

Modeling efforts were streamlined using graphical exploratory analyses of OS and PFS with the Kaplan–Meier method before building a base model. A non-parametric log-rank test was performed for the unadjusted OS and PFS analyses according to the treatment administered. Cox proportional hazards regression modeling, with treatment (SB8 and bevacizumab-EU) as an explanatory variable, was used to estimate hazard ratios and associated 95% CIs.

OS and PFS were described using a TTE modeling approach (Lim, 2021). Base models for OS and PFS without any covariates were created and selected first, and potential covariates were assessed thereafter. Various TTE models were tested, including exponential (Eq. 1), Weibull (Eq. 2), and log-logistic (Eq. 3) distributions of event times, and a proportional hazard model was used for the covariate effect. The final base models for OS and PFS were selected based on the lowest objective function value (OFV), graphical and statistical evaluations, and scientific plausibility.

$${S\;\left(t\right)=e}^{-\lambda t},$$ (1)

$$S\;\left(t\right)=e^{-{\left(\lambda t\right)}^{\gamma }},$$ (2)

$$S\;\left(t\right)\;{=\left\{1+{\left(\frac{t}{\lambda }\right)}^{\gamma }\right\}}^{-1},$$ (3)

where *S*(*t*) is a survival function, λ is the degree of decrease in *S*(*t*) over time, and γ is an additional parameter providing more flexibility in *S*(*t*) over time.

The goodness of fit of the models to the observed data was evaluated using statistical methods and the visual predictive check (VPC). The Kaplan–Meier plots of the observed data were summarized as median estimates and 95% CIs, and then, they were compared with the simulated medians and 95% prediction intervals (PIs) (2.5th–97.5th percentiles) determined using the respective models for OS and PFS. The VPC values were further stratified by quartiles of the observed bevacizumab C_ss,trough_. The Wald test was used for model evaluations, and the likelihood ratio test was used for comparing hierarchical models. A decrease of at least 3.84, corresponding to a *p*-value of 0.05, was considered a statistically significant improvement of fit during base model construction.

Covariate modeling was performed by the forward stepwise addition of covariates to the model, followed by a backward elimination procedure from the full model. In the univariate analysis, the statistical significance of each covariate was tested individually with a criterion for changes in an OFV of 6.63 (*α* = 0.01; df = 1). Covariates with the most significant decreases in the OFV were collectively set as a new base covariate model, and the process was repeated until there were no further covariates indicating a significant decrease in the OFV. If two or more covariates were included upon attainment of the full model, a more stringent OFV criterion of 7.88 (*α* = 0.005, df = 1) was applied to allow covariates to be maintained in the model.

The following covariates describing patient characteristics were selected for screening based on physiological plausibility and clinical relevance.

Age (<65 years vs. ≥65 years); sex (male vs. female); race (white vs. non-white); body weight (continuous); body mass index (BMI) (continuous); subtype of lung cancer (adenocarcinoma, large-cell neuroendocrine carcinoma, large-cell carcinoma, adenosquamous carcinoma, or other); Eastern Cooperative Oncology Group (ECOG) performance status at enrollment (0 vs. 1); smoking history (never smoker vs. former smoker vs. current smoker); treatment (SB8 vs. bevacizumab-EU); overall ADA (anti-drug antibody) results up to cycle 7 and overall ADA results by the end of treatment (positive vs. negative vs. inconclusive); pre-dose steady-state (continuous) concentrations of bevacizumab (SB8 and bevacizumab-EU); geographical region of residence (EU vs. non-EU); and country (Russia vs. Ukraine vs. Georgia; Hungary; Germany; Spain vs. Belarus; Romania; Serbia; Poland vs. Korea; Thailand; and Taiwan).

The demographic and clinical characteristics of the patients are summarized in Table 1.

**TABLE 1:** Demographic and clinical characteristics of patients included in the time-to-event analysis.

	SB8, *n* = 100	BEV-EU, *n* = 124	TotalN = 224
Categorical variables			
Sex			
Male, *n* (%)	58 (58)	73 (58.9)	131 (58.5)
Female, *n* (%)	42 (42)	51 (41.1)	93 (41.5)
Age			
<65 years, *n* (%)	67 (67)	88 (71)	155 (69.2)
≥65 years, *n* (%)	33 (33)	36 (29)	69 (30.8)
Race			
White, *n* (%)	94 (94)	114 (91.9)	208 (92.9)
Non-white, *n* (%)	6 (6)	10 (8.1)	16 (7.1)
Region			
EU, n (%)	31 (31)	26 (21)	57 (25.4)
Non-EU, *n* (%)	69 (69)	98 (79)	167 (74.6)
Country group			
Belarus, Romania, Serbia, and Poland, *n* (%)	29 (29)	30 (24.2)	59 (26.3)
Georgia, Hungary, Germany, and Spain, *n* (%)	20 (20)	20 (16.1)	40 (17.9)
Korea, Thailand, and Taiwan, *n* (%)	6 (6)	10 (8.1)	16 (7.1)
Russia, *n* (%)	25 (25)	34 (27.4)	59 (26.3)
Ukraine, *n* (%)	20 (20)	30 (24.2)	50 (22.3)
Smoking status			
Non-smoker, *n* (%)	43 (43)	48 (38.7)	91 (40.6)
Former smoker, *n* (%)	23 (23)	35 (28.2)	58 (25.9)
Current smoker, *n* (%)	34 (34)	41 (33.1)	75 (33.5)
Cancer type			
Adenocarcinoma, *n* (%)	97 (97)	112 (90.3)	209 (93.3)
Large-cell neuroendocrine carcinoma, *n* (%)	0 (0)	1 (0.8)	1 (0.4)
Large-cell carcinoma, *n* (%)	0 (0)	4 (3.2)	4 (1.8)
Adenosquamous carcinoma, *n* (%)	0 (0)	1 (0.8)	1 (0.4)
Not otherwise specified, *n* (%)	3 (3)	6 (4.8)	9 (4)
Stage			
IB, *n* (%)	0 (0)	1 (0.8)	1 (0.4)
IV, *n* (%)	100 (100)	123 (99.2)	223 (99.6)
Baseline ECOG status			
0, *n* (%)	35 (35)	39 (31.5)	74 (33)
1, *n* (%)	65 (65)	85 (68.5)	150 (67)
Overall ADA by cycle 7			
Positive, *n* (%)	16 (16)	15 (12.1)	31 (13.8)
Negative, *n* (%)	81 (81)	103 (83.1)	184 (82.1)
Inconclusive, *n* (%)	3 (3)	5 (4)	8 (3.6)
Missing, n (%)	0 (0)	1 (0.8)	1 (0.4)
Overall ADA by the end of treatment			
Positive, *n* (%)	20 (20)	16 (12.9)	36 (16.1)
Negative, *n* (%)	78 (78)	102 (82.3)	180 (80.4)
Inconclusive, *n* (%)	2 (2)	5 (4)	7 (3.1)
Missing, *n* (%)	0 (0)	1 (0.8)	1 (0.4)
Continuous variables			
Trough concentration (µg/mL), median (Q1–Q3)	115.1 (87.8–144.6)	125.5 (97.0–153.6)	119.5 (92.0–150.5)
Weight (kg), median (Q1–Q3)	71.3 (64.0–83.0)	73 (65.0–82.0)	72.1 (64.2–82.0)
Body mass index (kg/m^2^), median (Q1–Q3)	25.35 (23.2–28.4)	25.4 (22.8–29.1)	25.4 (23–28.7)

ADA, anti-drug antibody; BEV-EU, bevacizumab-EU; ECOG, Eastern Cooperative Oncology Group; EU, European Union; Q1: first quartile, Q3: third quartile.

The overall ADA results were determined as “positive” for a patient with treatment-induced or treatment-boosted ADA, where treatment-induced ADA indicates at least one positive result after pre-dose of cycle 1 for patients with negative ADA at pre-dose of cycle 1 and treatment-boosted ADA indicates at least one positive result with a higher titer level compared to the pre-dose of cycle 1 after pre-dose of cycle 1 for patients with positive ADA at pre-dose of cycle 1.

A proportional hazard model was implemented to test each of the covariates on the baseline hazard. C_ss,trough_ was taken from the pre-dose serum concentrations of SB8 and bevacizumab-EU measured mostly before cycle 7 (221 samples) or else cycle 5 (3 samples) at ≥100 days after the first dosing in each patient (Roche Registration GmbH, 2005). To describe the drug’s concentration effect on survival or disease progression in terms of fractional decrease of the hazard, an inhibitory sigmoidal maximum effect (E_max_) model (Eq. 4) was implemented as follows (Chigusta et al., 2017):

$${H\left(t\right)=H}_{0}\left(t\right)\left[1-\left(\frac{E_{\max}\;*\;{C_{ss,trough}}^{Hill}}{{{EC}_{50}}^{Hill}+{C_{ss,trough}}^{Hill}}\right)\right],$$ (4)

where *H*(*t*) and $H_{0}$ (*t*) are the hazard and baseline hazard, respectively; $C_{ss,trough}$ is the steady-state trough concentration of treatment; $E_{\max}$ is the maximum inhibitory effect of treatment on the hazard; ${EC}_{50}$ is $C_{ss,trough}$ at half of $E_{\max}$ ; and Hill is the hill coefficient.

For categorical covariates with missing data, a mixture model was applied in NONMEM^®^, version 7.4 (ICON Development Solutions, Ellicott City, MD, United States), to impute missing data based on weighted maximum likelihood (Frame et al., 2003). In the mixture model, the missing proportion was fixed at the respective proportion of the observed data under the assumption that the data were missing completely at random.

### Simulation study for the exposure–response relationship

The exposure–response curve for OS and PFS by the range of C_ss,trough_ was simulated by implementing inverse functions for the TTE functions in NONMEM codes for the respective final models.

To further determine concentrations corresponding to 50% of the simulated maximum median time to event, where the event is death for OS and disease progression or death for PFS (hereafter abbreviated as TTE50CONC), 50% of the maximum median TTE was first calculated from the half of the difference between the simulated baseline median TTE and the maximum median TTE (plus the baseline median TTE value) estimated using the final models for OS and PFS. The median TTE associated with concentrations ranging from 0 to 150 μg/mL was tested at increments of 0.1 μg/mL during simulation. The conditional statement in the $ERROR block in the NM-TRAN control records allowed the tested concentration to be capped if the response associated with that concentration reached the half-maximal response in the exposure–response curve (Supplementary Table S1). This way, the largest concentration approaching the half-maximal response was determined to be TTE50CONC. Similarly, concentrations corresponding to 90% of the simulated maximum median TTE for OS and PFS (hereafter abbreviated as TTE90CONC) were derived using the same approach to assess 90% of the maximal response.

**FIGURE 1:**
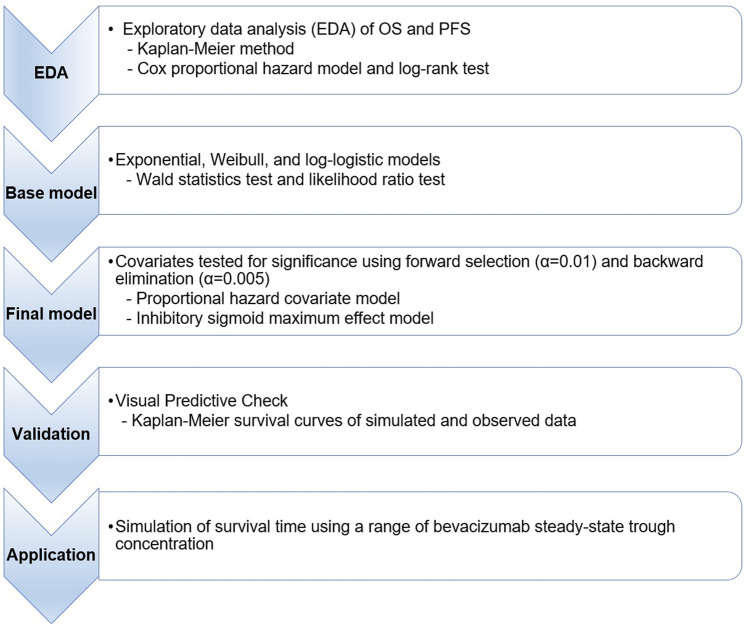
Workflow for time-to-event simulation modeling and the analysis of overall survival and progression-free survival. OS, overall survival; PFS, progression-free survival.

An overview of the workflow for this modeling and simulation is provided in Figure 1.

All model development and parameter estimations throughout this study were performed by first-order conditional estimation with the interaction method using NONMEM. Data handling, graphical model diagnosis, and statistical analyses were performed using R, version 4.2.3 (R Foundation for Statistical Computing, Vienna, Austria).

## Results

**FIGURE 2:**
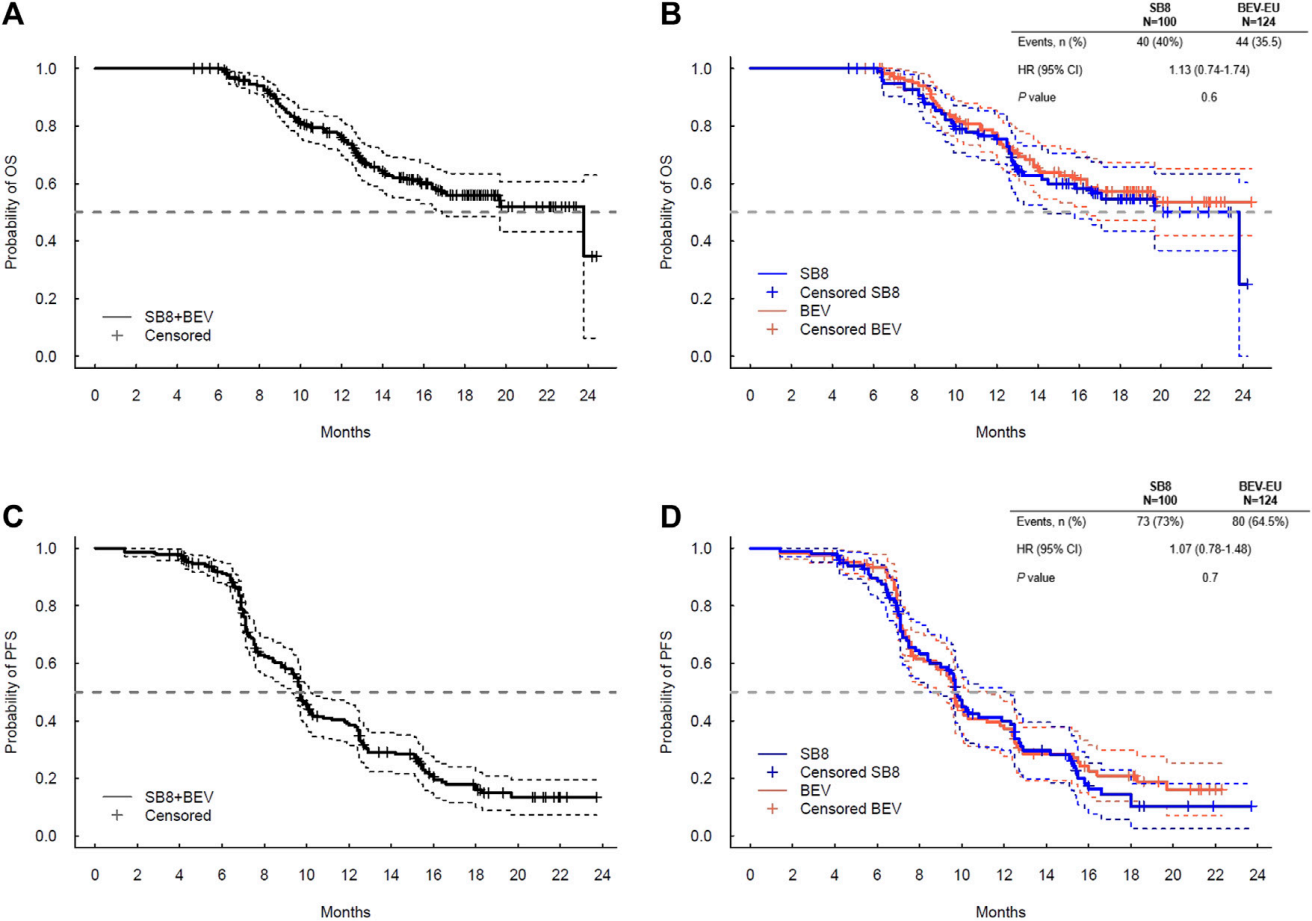
Kaplan–Meier plot for **(A)** overall survival for SB8 + BEV-EU; **(B)** overall survival by the treatment group; **(C)** progression-free survival for SB8 + BEV-EU; and **(D)** progression-free survival by Cox proportional hazard model results for OS and PFS analyzed by SB8 and bevacizumab-EU treatment groups. BEV, reference EU-sourced bevacizumab; HR, hazard ratio; OS, overall survival; and PFS, progression-free survival.

The exploratory analyses for OS and PFS using the combined data (SB8 + bevacizumab-EU) and separate data by the treatment group show comparable survival curves between SB8 and bevacizumab-EU (Figure 2). The log-rank test revealed no treatment differences in terms of both OS (*p* = 0.6) and PFS (*p* = 0.6), and the estimated hazard ratios were 1.13 (95% CI: 0.74–1.74; *p* = 0.6) for OS and 1.07 (95% CI: 0.78–1.48; *p* = 0.7) for PFS.

**FIGURE 3:**
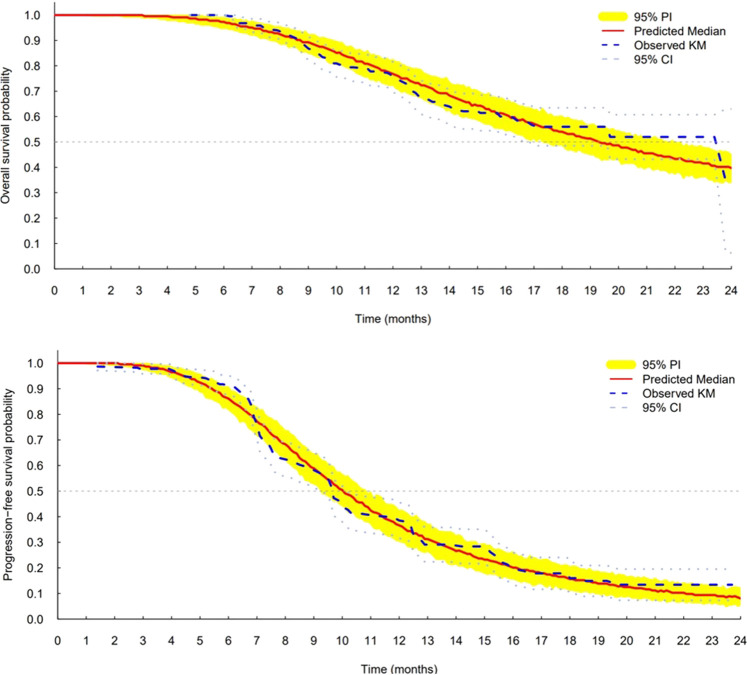
Visual predictive check plots for final models of overall survival (top) and progression-free survival (bottom). CI, confidence interval; KM, Kaplan–Meier; and PI, prediction interval.

**FIGURE 4:**
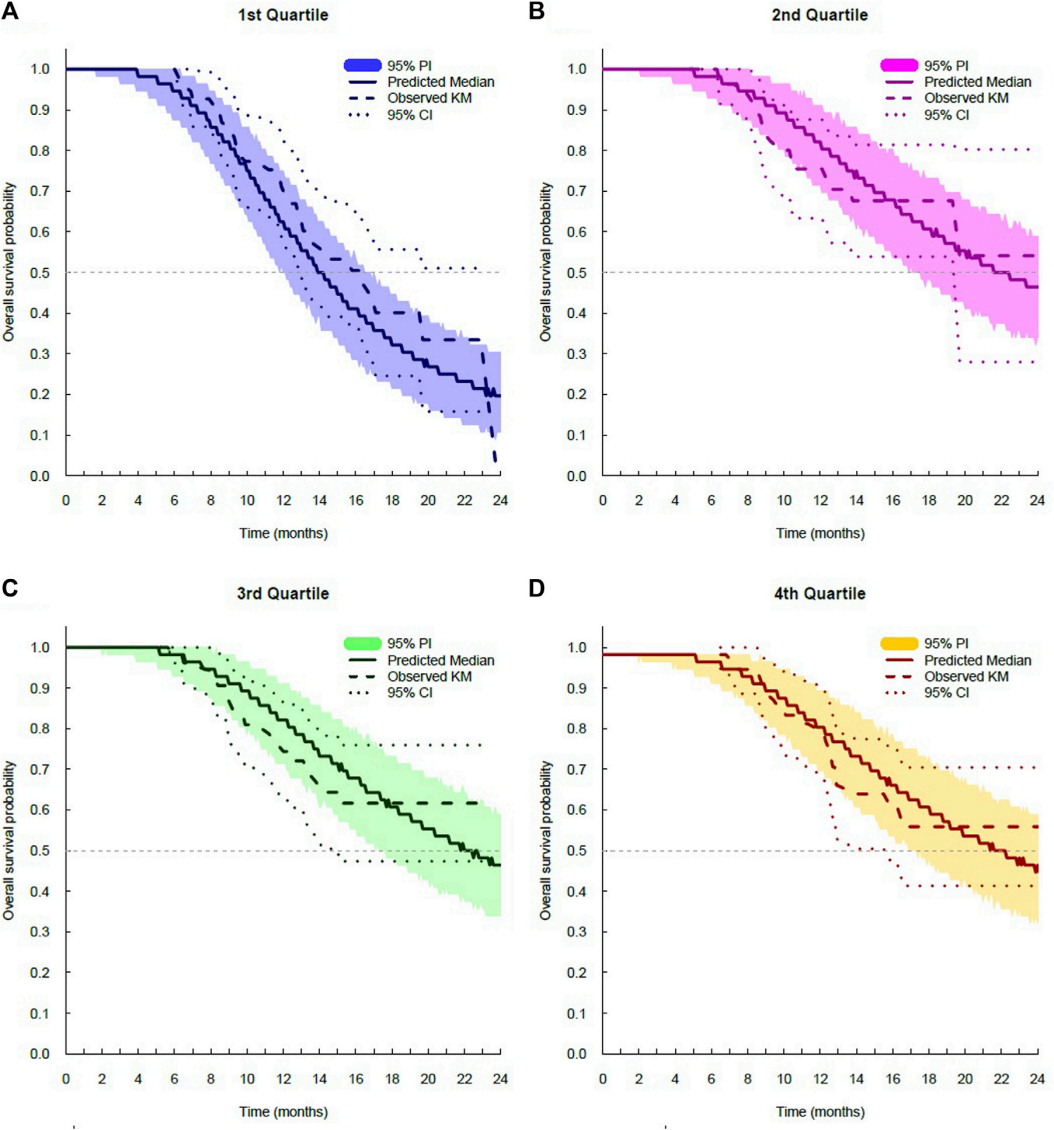
Visual predictive check plots for overall survival stratified by exposure quartiles: **(A)** C_ss,trough_ quartile 1; **(B)** C_ss trough_ quartile 2; **(C)** C_ss,trough_ quartile 3; and **(D)** C_ss,trough_ quartile 4. CI, confidence interval; KM, Kaplan–Meier; and PI, prediction interval.

**FIGURE 5:**
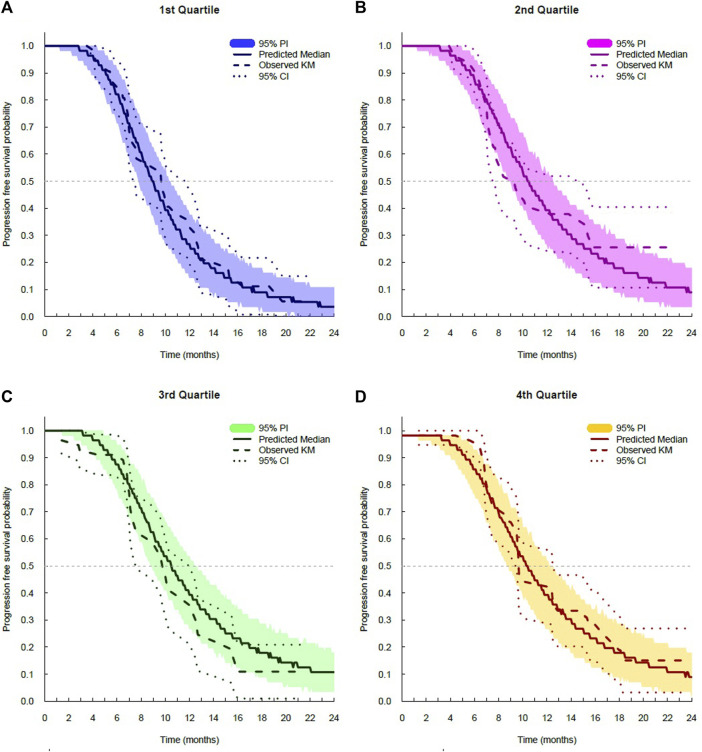
Visual predictive check plots for progression-free survival stratified by exposure quartiles: **(A)** C_ss,trough_ quartile 1; **(B)** C_ss,trough_ quartile 2; **(C)** C_ss,trough_ quartile 3; and **(D)** C_ss,trough_ quartile 4. CI, confidence interval; KM, Kaplan–Meier; and PI, prediction interval.

The log-logistic distribution model best described the time to OS and PFS. Based on the log-likelihood ratio test, the Weibull distribution model was superior to the exponential distribution model in all cases. The log-logistic distribution model had the lowest OFV for both OS and PFS. Although testing for statistical significance between Weibull and log-logistic was not applicable since these models do not have a full-nested relationship, the predictive performance for OS and PFS was similar between the log-logistic and Weibull models, as evidenced by VPC plots. The VPC results show the 95% PI of the simulated data overlaid on the Kaplan–Meier curve of the observed data, indicating that the model predictions aligned well with the observed data (Figure 3). The median, 95% PI, and 95% CI largely overlapped each other on VPC results for OS and PFS stratified by quartiles of bevacizumab C_ss,trough_ (Figure 4, Figure 5).

C_ss,trough_ was a significant predictor of the hazard for OS and PFS. Covariate analysis did not indicate any other patient demographic or clinical characteristics to be significant enough to be included in the final model (Table 2). In the univariate analysis for the OS model, BMI, weight, and C_ss_,_trough_ were significant based on the criterion of *p* = 0.01 (ΔMOFV = −6.63), but BMI and weight did not meet the criteria during the forward selection procedure and, thus, were not included in the full model. In the univariate analysis for the PFS model, only C_ss,trough_ was significant based on the criterion of *p* = 0.01 (ΔMOFV = −6.63) and was, thus, included in the final model. Treatment (SB8 vs. bevacizumab-EU) did not affect the models for OS and PFS. C_ss,trough_ was not a significant covariate for any model when a proportional hazard model was used, but when a sigmoid maximum effect model was implemented, C_ss,trough_ significantly improved the model. The Hill coefficient in the sigmoid maximum effect model was tested with a range of 1–100, and a value of 20 was fixed in the model. The selected value of the Hill coefficient suggests a nearly all-or-none effect of SB8 and bevacizumab-EU on OS and PFS. The C_ss,trough_ values at half of E_max_ (EC_50_) for the OS and PFS models were estimated as 77.5 and 77.7 μg/mL, respectively. The final TTE models for both OS and PFS are described in Table 3.

**TABLE 2:** Model selection for the time-to-event analysis.

	Overall survival		Progression-free survival	
Model description	OFV	ΔOFV	OFV	ΔOFV
Base model (no covariate)	709.412	—	991.293	—
+Age	709.283	−0.129	990.845	−0.448
+Sex	707.685	−1.727	988.308	−2.985
+Race	705.362	−4.05	991.050	−0.243
+Weight	700.456	−8.956*	988.471	−2.822
+BMI	701.799	−7.613*	987.978	−3.315
+Region	708.651	−0.761	987.905	−3.388
+Country groups	704.252	−5.16	987.903	−3.390
+Smoking status	709.373	−0.065	990.720	−0.573
+Cancer type	709.412	0	988.921	−2.372
+Baseline ECOG	708.724	−0.688	988.196	−3.097
+Concentrations	689.951	−19.461*	982.600	−8.693*
+Overall ADA by cycle 7	709.248	−0.164	990.475	−0.818
+Overall ADA by EOT	707.670	−1.742	990.571	−0.722
+Treatment (SB8 vs. EU Avastin^®^)	709.347	−0.065	991.288	−0.005
New base model (with concentrations)	689.951	—		
+Weight	684.632	−5.319		
+BMI	685.536	−4.415		
+Concentrations**	689.951	−19.461***	982.600	−8.693***

A stepwise addition (*p* = 0.01, ΔOFV = −6.63) and elimination (*p* = 0.005, ΔOFV = −7.88) method was applied in the selection of covariates.

*Covariate showing statistical significance based on *p*= 0.01 (ΔOFV = −6.63) in the univariate analysis.

**The final models for overall survival and progression-free survival.

***–19.461 is the difference in objective function values between the base model for overall survival and the final model with concentrations, and −8.693 is the difference in objective function values between the base model for progression-free survival and the final model with concentrations.

BMI, body mass index; OFV, objective function value; ΔOFV, change in the OFV relative to the preceding model; ADA, anti-drug antibody; ECOG, Eastern Cooperative Oncology Group; EOT, end of treatment; EU, European Union.

**TABLE 3:** Parameter estimates in the final models for overall survival and progression-free survival.

	Parameter	Estimates	RSE (%)	95% CI
Overall survival	λ	12.0	4.13	11.028–12.972
	γ	3.67	3.13	3.445–3.895
	E_MAX_	0.704	3.98	0.649–0.759
	EC_50_ (μg/mL)	77.5	2.80	73.247–81.753
	HILL	20	—	—
Progression-free survival	λ	8.28	7.97	6.986–9.574
	γ	3.94	9.19	3.230–4.650
	E_MAX_	0.451	28.82	0.196–0.706
	EC_50_ (μg/mL)	77.7	5.08	69.958–85.442
	HILL	20	—	—

λ and γ are scale and shape factors, respectively, in the log-logistic model.

E_max_, maximum inhibitory effect; EC_50_, steady-state trough concentrations at half of E_max_; HILL, Hill coefficient in the sigmoid maximum effect model; RSE%, relative standard error; CI, confidence interval.

**FIGURE 6:**
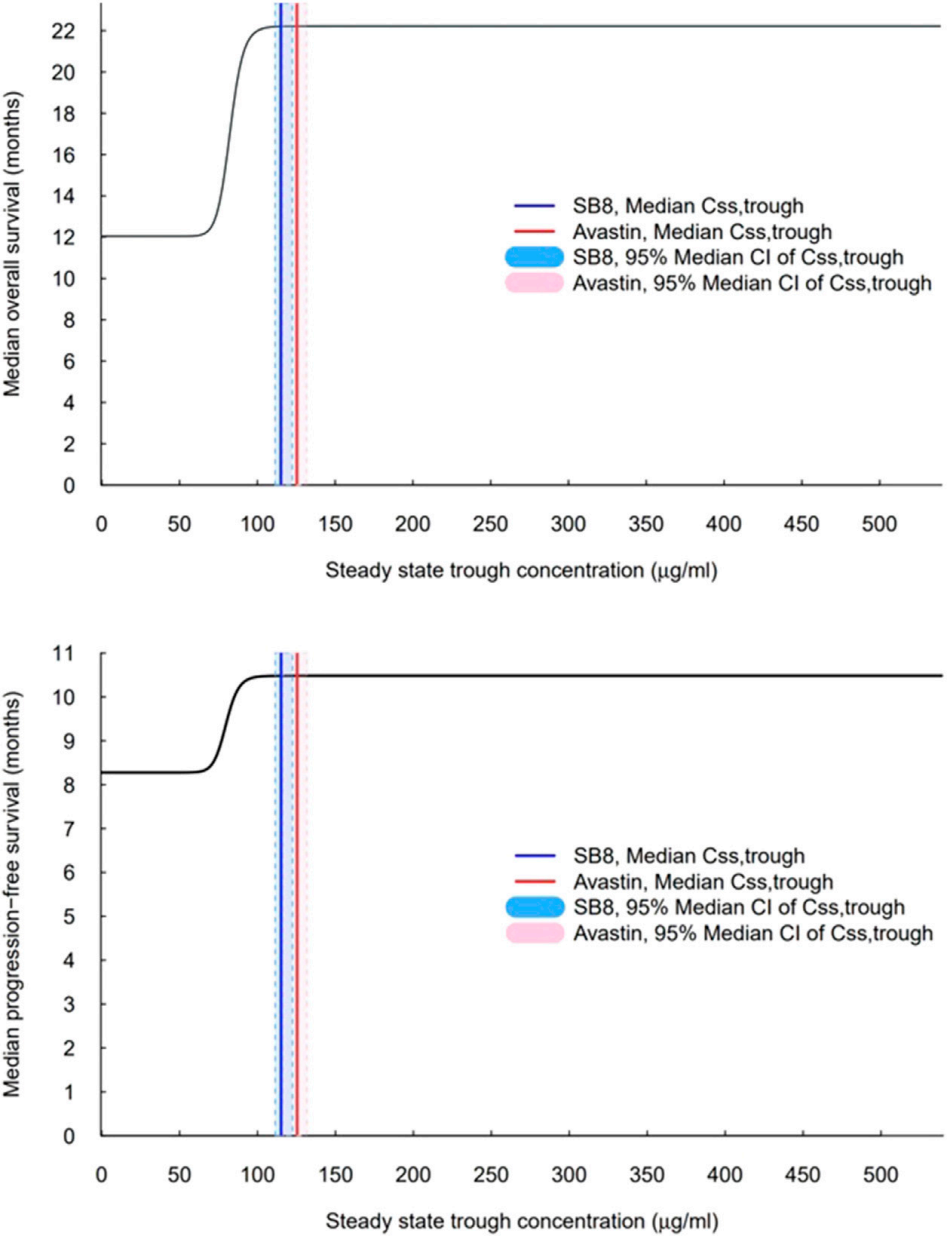
Exposure–response curves for overall survival and progression-free survival based on the final models. The vertical solid lines indicate observed median steady-state trough concentrations (C_ss,trough_) for SB8 (blue) and bevacizumab-EU (red) at 15 mg/kg intravenous infusion every 3 weeks. The shaded areas indicate the 95% median CI of steady-state trough concentrations for SB8 (light blue) and bevacizumab-EU (pink).

Simulations of median OS and PFS by ranges of C_ss,trough_ were carried out using the constructed final TTE models. A steep exposure–response relationship was shown, wherein the predicted median OS and PFS rise rapidly with increasing C_ss,trough_, plateau after a certain value (i.e., the inflection point), and then, remain unchanged with further increases in C_ss,trough_ (Figure 6). When comparing the interquartile range of C_ss,trough_ to the exposure–response curve, the lower end of the range for both treatments was on the rising curve, while the upper range of concentrations was at a plateau. The median C_ss,trough_ values of SB8 (115.114 μg/mL) and bevacizumab-EU (125.453 μg/mL) were observed from the clinical study of the simulations; both treatments were well above the steep part of the exposure–response curves, and the 95% bootstrap median CIs of C_ss,trough_ for SB8 and bevacizumab-EU overlapped one another on the plateaus. The 50th and 90th percentiles of the predicted median OS were 17.1 months and 21.2 months, respectively. The 50th and 90th percentiles of the predicted median PFS were 9.38 months and 10.3 months, respectively. Based on these values, the TTE50CONC and TTE90CONC values derived from simulation were 82.4 and 92.2 μg/mL, respectively, for OS and 79.7 and 89.1 μg/mL, respectively, for PFS.

## Discussion

This simulation study provided a unique modeling and simulation framework for characterizing the relationship between bevacizumab C_ss,trough_ and its efficacy in terms of OS and PFS in advanced NSCLC. To our knowledge, this is the first study that proceeded to publication after the conduct of a TTE model-based simulation approach and evaluation of the efficacy of a bevacizumab biosimilar along with a reference biologic based on the concentration–response (time to death and time to disease progression) relationship.

TTE modeling was performed using OS and PFS data for patients with metastatic or recurrent non-squamous NSCLC who had available pre-dose steady-state concentration data from a comparative clinical efficacy and safety study. Covariate analysis evaluated the impact of key patient-level factors on survival or disease progression. The final models for both OS and PFS were selected, with C_ss,trough_ as a covariate showing sigmoidal maximum effects. Simulation to assess exposure in relation to median OS and PFS was carried out by implementing the inverse functions for median TTE functions in the respective final models. The bevacizumab exposure–response curve for OS and PFS based on the corresponding final model reflects a steep relationship where the median TTE (OS or PFS) corresponds with a phase of sharp increase, resulting in a wide range of bevacizumab concentrations showing maximum efficacy.

Clinical factors affecting the survival of patients with advanced NSCLC are worth mentioning. Through the univariate analysis of covariates, we noted that the hazard for OS increased with decreasing BMI and weight; however, this increase was not statistically significant. This observation was consistent with previous findings of low BMI and significant weight loss (≥5%) as related factors associated with poor survival among patients with NSCLC (Hoang et al., 2012; Shepshelovich et al., 2019). Weight-based bevacizumab dosing for the treatment of solid cancer types, accounting for the body weight’s contribution to clearance and volume of distribution, may explain these results (Lu et al., 2008; Roche Registration GmbH, 2023). Nevertheless, the association between BMI and survival outcomes with bevacizumab treatment (along with other patient factors interacting with BMI affecting poor prognosis) remains to be fully elucidated. Furthermore, the impact of overall positive ADA results on OS and PFS showed no significance in our model, and in the comparative phase III study, ADA formation at each time point of the cycle was comparable between SB8 and bevacizumab-EU (Samsung Bioepis NL B.V, 2023b). It is to be noted that the clinical significance of anti-bevacizumab is not known (FDA, 2022). In neither of the covariate analyses for OS and PFS did the treatment (SB8 or bevacizumab-EU) affect hazards differently, suggesting that the treatment effects on OS and PFS were comparable between SB8 and bevacizumab-EU.

In the final model, an E_max_ model was used to empirically describe hazard reduction according to a range of concentrations (C_ss,trough_). While this model may be limited in its physiological basis, the relationship between the concentration of a drug and its pharmacological effects, whether the variable is binary or continuous, can be conveniently described by a sigmoid E_max_ model, especially in cases where the relationship is close to that of a cumulative log-normal distribution (Bowling et al., 2009; Proost et al., 2020). In finding the best-fit model, a wide range of Hill coefficients was tested, from a simple E_max_ model (*γ* = 1) to a steep model (*γ* = 100), to describe the drug’s pharmacological effect. This way, a proper estimation of parameters concerning pharmacological efficacy (E_max_) and potency (EC_50_) was obtained to determine the hazard function according to drug concentrations. When a hazard was determined based on a given concentration value in the final TTE model, the median OS and PFS were subsequently determined from the corresponding hazard, and TTE50CONC and TTE90CONC were further determined from simulation to provide clinical context to the hazard.

As the last step in model development, the TTE VPC was used to evaluate model performance, showing that the final models for OS and PFS fit well with the observed survival data in the Kaplan–Meier curves. However, around year 2, the model predicted a slightly lower median survival value than the observed median values. Nonetheless, the 95% CI for the observed values overlapped with the 95% PI throughout the observation period, indicating no statistical significance. Also, the low number of patients in the data at this time point influenced diagnostic accuracy and precision.

The simulated exposure–response curve aligns well with the response associated with steady-state concentrations measured following multiple doses of bevacizumab at 7.5 and 15 mg/kg, as reported in historical investigations of the reference product, Avastin^®^ (FDA, 2004; Johnson et al., 2004; FDA, 2006). With C_ss,trough_ between 73 ± 43 μg/mL and 135 ± 48 μg/mL reported for the respective doses of 7.5 and 15 mg/kg of Avastin^®^ in a phase II NSCLC study (AVF0757g), an optimal therapeutic response may be seen in many patients with a dose of 7.5 mg/kg; concentrations within this range are right at or above those located at the rapid increase of the median TTE curve. Moreover, when concentrations are increased with a higher dose at 15 mg/kg, the response associated with the upper end of the reported concentration range (i.e., 135 μg/mL) would show the maximum efficacy of bevacizumab based on the exposure–response curves. Similarly, the interquartile range of C_ss,trough_ (92.0–150.5 μg/mL) for SB8 and bevacizumab-EU was associated with the rising and plateau of the exposure–response curve, indicating therapeutic response. These comparisons show that the modeling and simulation accurately and predictably characterized the concentration-dependent response of bevacizumab within the therapeutic range.

In addition to visual representations of the exposure–response relationship, the shape of the curve was further explored to understand the range of relevant values by computing the concentrations corresponding to 50% and 90% of the maximum median TTE (TTE50CONC, TTE90CONC) for OS and PFS, respectively. The median and interquartile range of median C_ss,trough_ of both SB8 and bevacizumab-EU, along with the previously reported range for C_ss,trough_ of Avastin^®^, were all well above TTE50CONC. While other methods, such as the use of inverse functions of the simulation codes in the final model, may be feasible for estimation, this requires the analytical calculation of each relevant parameter in the TTE function and Hill equations to derive the final value. Thus, in our analyses, conditional statements were used to find concentrations associated with the response of interest and then to cap the values once they were determined. For this purpose, the simulation dataset was prepared with a narrow concentration interval ranging from zero to the maximum bevacizumab C_ss,trough_ reported in the clinical study, in increments of 0.1 μg/mL, enabling NONMEM to compute TTE50CONC and TTE90CONC with reasonable accuracy.

Our modeling analyses based on the principle of TTE have utility in evaluating the comparative efficacy of two treatments. The analyses remain empirical and exploratory for the purpose of predicting the effect of dose changes and dose optimization, as the model is based on clinical data for monoclonal antibodies tested at a single-dose level with available C_ss,trough_. Further improvement can be made by applying a mixed-effects analytical approach. First, the effects of inter-individual variabilities of bevacizumab PK were not incorporated, and thus, typical prediction of TTE outcomes was made from the current final model. Estimating and incorporating the inter-individual variability of bevacizumab PK into TTE models help elicit valuable insights into a predictive TTE response interval in relation to drug exposure, which in turn provides more biologically plausible predictions for OS and PFS based on individual plasma concentration levels. Also, studies using PK/PD modeling may discern additional concentration-dependent covariates and time-varying PK parameters (e.g., time-varying clearance with tumor dynamics), potentially affecting the final TTE models for OS and PFS (Liu et al., 2017). Thus, given the sensitivity of tumor size to monoclonal antibodies used in oncology and the association between tumor growth rate and survival in the context of advanced NSCLC (Claret et al., 2009; Wang et al., 2009; Boyle, 2010; Gong et al., 2020), it would be worth assessing the impact of changes in tumor size after bevacizumab treatment. In particular, a separate model for predicting the tumor size can be developed to integrate model-predicted changes in the tumor size into the survival model, and the current model may be updated based on early readouts related to tumor size. Ultimately, such analysis based on tumor size-integrated TTE modeling, along with modeling and simulation results such as those generated by our study, could guide in predicting outcomes associated with the same classes of anti-VEGF drugs using early readouts on tumor size.

From the perspective of biosimilarity evaluation, our study extended the application of PK/PD modeling and simulation to evaluate the comparative efficacy of a biosimilar and its reference biologic based on their exposures (Wang et al., 2019). In a biosimilar development program, modeling and simulation are typically applied to optimize study design, ensuring adequate sensitivity in the selected study population, appropriate dosing, and sufficient sample size based on an understanding of inter-individual variability in PK/PD (Zhu et al., 2018; Li et al., 2020). In addition, a quantitative relationship between a PD biomarker relevant to a drug’s mechanism of action, as well as clinical endpoints, can be assessed through the PK/PD model, providing the information necessary to assess the model’s utility as a surrogate primary endpoint in clinical research (Lee et al., 2011; Li L et al., 2018; Jang et al., 2022). In cases where limited comparative data are available for biosimilars, network meta-analysis can be performed to assess the similarity of the products (Chiumente et al., 2017), and such analysis may be conducted through modeling as well. In addition to these well-known, conventional applications, our exposure–response analysis using a TTE model provides a novel “science-applied tool” for assessing the potential impact of exposure differences between a biosimilar and the reference product on clinical efficacy.

In conclusion, our modeling and simulation analysis elucidated the exposure–response relationship of bevacizumab in the treatment of patients with advanced NSCLC, providing fundamental information about the drug. This relationship further substantiates the similarity in treatment effects between SB8 and EU-bevacizumab.

## Data Availability

The original contributions presented in the study are included in the article/Supplementary Material; further inquiries can be directed to the corresponding author.
