# Supplementary material for: Exposure–response analysis using time-to-event data for bevacizumab biosimilar SB8 and the reference bevacizumab
Source: Front Pharmacol. 2024 Jan 16;14:1288308. doi: 10.3389/fphar.2023.1288308 (PMC10825021; doi:10.3389/fphar.2023.1288308)
Supplement: Supplementary file 1 [file Table1.DOCX]

Supplementary Material

**NONMEM control file**

$PROB Overall survival, SB8 and bevacizumab-EU

$INPUT ID TIME DV CONC MDV

$DATA OS_simul.csv IGNORE=@

$SUBR ADVAN=13 TOL=6

$ABB COMRES = 2

$MODEL

COMP=(HAZARD)

$PK

IF(NEWIND.LE.1) THEN

SURV = 1

ENDIF

LAMDA = THETA(1)*EXP(ETA(1))

GAMMA = THETA(2)

EMAX = THETA(3)

EC50 = THETA(4)

HILL = THETA(5)

EFF = EMAX*(CONC**HILL)/((EC50**HILL)+(CONC**HILL))

DEL = 0.000001

IF(NEWIND.EQ.0) THEN

COM(1) = -1

COM(2) = -1

ENDIF

$DES

H1 = (GAMMA/LAMDA)*((T+DEL)/LAMDA)**(GAMMA-1)

H2 = 1 + ((T+DEL)/LAMDA)**GAMMA

HAZ = (H1/H2)*(1-EFF)

DADT(1) = HAZ

$ERROR

CUMHAZ = A(1)

SURV= EXP(-CUMHAZ)

IF (DV.EQ.1) THEN

Y = SURV

ELSE

HAZNOW = (((GAMMA/LAMDA)*((TIME+DEL)/LAMDA)**(GAMMA- 1))/(1+((TIME+DEL)/LAMDA)**GAMMA) 
*(1-EFF)

Y = SURV*HAZNOW

ENDIF

MEDTTE = EXP((LOG(EXP(-LOG(2)/(EFF-1))-1)/GAMMA)+LOG(LAMDA)) ; Median Time to Event

BMEDTTE = EXP((LOG(EXP(-LOG(2)/(0-1))-1)/GAMMA)+LOG(LAMDA)) ; Baseline Median TTE

MAXMEDTTE = EXP((LOG(EXP(-LOG(2)/(EMAX-1))-1)/GAMMA)+LOG(LAMDA)) ;Maximum Baseline TTE

MAXMEDTTED = MAXMEDTTE - BMEDTTE

MEDTTE50 = 0.5*MAXMEDTTED + BMEDTTE ;50% of maximum median TTE

MEDTTE90 = 0.9*MAXMEDTTED + BMEDTTE ;90% of maximum median TTE

IF(MEDTTE.LE.MEDTTE50) THEN

COM(1) = CONC

ENDIF

IF(MEDTTE.LE.MEDTTE90) THEN

COM(2) = CONC

ENDIF

EC50MEDTTE = COM(1) ;TTE50CONC

EC90MEDTTE = COM(2) ;TTE90CONC

$MSFI =304.MSF

$SIM (20230629) ONLYSIM TRUE=FINAL NSUB=1

$TABLE ID CONC MEDTTE REP BMEDTTE MAXMEDTTE MAXMEDTTED MEDTTE50 MEDTTE90 EC50MEDTTE EC90MEDTTE

NOAPPEND NOPRINT FILE=OS_304_ER_simul.txt ; NOHEADER
